# Standardized Procedures Important for Improving Low-Temperature Ceramic Fuel Cell Technology: From Transient to Steady State Assessment

**DOI:** 10.3390/nano11081923

**Published:** 2021-07-26

**Authors:** Fan Yang, Yifei Zhang, Jingjing Liu, Muhammad Yousaf, Xinlei Yang

**Affiliations:** 1 Jiangsu Provincial Key Laboratory of Solar Energy Science and Technology, School of Energy & Environment, Southeast University, Nanjing 210096, China; YFZH147@163.com (Y.Z.); liujingjing_970812@163.com (J.L.); tahayousaf@gmail.com (M.Y.); brobow@163.com (X.Y.); 2 Key Laboratory of Energy Thermal Conversion and Control of Ministry of Education, School of Energy & Environment, Southeast University, Nanjing 210096, China

**Keywords:** standardized procedure, transient state power, quasi-steady-state performance, semiconductor ionic fuel cell, semiconductor membrane fuel cell

## Abstract

As the stress–strain curve of standardized metal samples provides the basic details about mechanical properties of structural materials, the polarization curve or current–voltage characteristics of fuel cells are vitally important to explore the scientific mechanism of various solid oxide cells aiming at low operational temperatures (below 600 °C), ranging from protonic conductor ceramic cells (PCFC) to emerging Semiconductor ionic fuel cell (SIFC)/Semiconductor membrane fuel cells (SMFC). Thus far, worldwide efforts to achieve higher nominal peak power density (PPD) at a low operational temperature of over 0.1 s/cm ionic conductivity of electrolyte and super catalyst electrode is the key challenge for SIFCs. Thus, we illustrate an alternative approach to the present PPD concept and current–voltage characteristic. Case studies reveal that the holy grail of 1 W/cm^2^ from journal publications is expected to be reconsidered and normalized, since partial cells may still remain in a transient state (TS) to some extent, which means that they are unable to fulfill the prerequisite of a steady state (SS) characteristic of polarization curve measurement. Depending on the testing parameters, the reported PPD value can arbitrarily exist between higher transient power density (TPD) and lower stable power density (SPD). Herein, a standardized procedure has been proposed by modifying a quasi-steady state (QSS) characterization based on stabilized cell and time-prolonged measurements of common *I*–*V* plots. The present study indicates, when compared with steady state value, that QSS power density itself still provides a better approximation for the real performance of fuel cells, and concurrently recalls a novel paradigm transformation from a transient to steady state perspective in the oxide solid fuel cell community.

## 1. Introduction

Searching oxide ion-conducting oxide materials with a low operating temperature of the solid oxide fuel cell (SOFC) has become the mainstream approach, owing to economic and technical demands [1]. Over the past decade, scientific and engineering efforts have been facilitated by the development of electrolytes with >0.1 s/cm ionic conductivity and a super catalyst electrode, together with new cell structure and improved fabrication techniques for the commercialization of low-temperature SOFCs [2]. Until now, remarkable achievements have been demonstrated at low operational temperatures of constructed cells, wherein a peak power density of 1700 mW/cm^2^ at 650 °C [3] has been observed in the lab-scale cell under hydrogen fuel (H_2_), as well the oxidizing atmosphere (air) of carbon dioxide/oxygen mixture gas. Moreover, a giant power output of about 2 W/cm^2^ with reliable durability measurements at 550 °C were able to be achieved in the constructed fuel cell device because of advanced nano-engineering cathode materials [4,5]. For commercialization, cell stacks as a product on doped ceria GDC was well demonstrated in the recent SOFC Market, exhibiting a reliable fuel cell performance of 0.2%/1000 h degradation below 600 °C [6].

As reported in SOFC literature, a large quantity of recorded nominal peak power density (PPD) has been achieved (200–1000 mW/cm^2^) below 600 °C in SOFC [7,8]. Moreover, semiconductor ionic fuel cell (SIFC)/semiconductor membrane fuel Cells (SMFC), exhibit the super-fast ion transportation through the surface/interface and heterostructure based semiconducting oxide materials constructed as an electrolyte functional layer (EFL). In addition, the energy band offsetting and electron-ion coupling effect between ion-conductor/semiconductors contribute to attaining feasible electro-chemical catalytic activity and low polarization resistance in the fuel cell environment. The achieved power output from the various SIFC/SMFCs mechanism is rather diverse, but the general trend is that the reported PPD of cells can be increased at lower operating temperatures. Xia Chen et al. reported BCFZY-ZnO system, which exhibits an excellent PPD of 643 mW/cm^2^ at 500 °C [9]; likewise, the heterostructure of LCP-ZnO also displays 864 mW/cm^2^ at 550 °C [10]. In SOFCs, the electron leakage and durability measurement of semiconductor materials are still main concerns.

**Figure 1:**
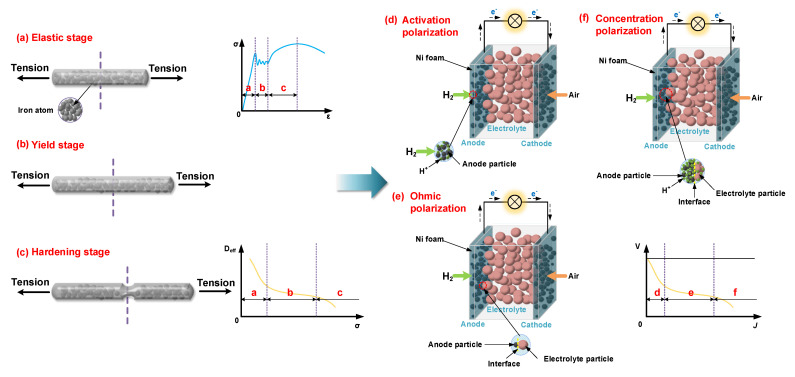
Stress–strain process of metal samples: (**a**) elastic stage, (**b**) yield stage, (**c**) hardening stage; polarization process of fuel cells: (**d**) activation polarization, (**e**) ohmic polarization, (**f**) concentration polarization.

In SOFC, the maximum/peak power density (MPD/PPD) of constructed cells can be acquired directly from the polarization curve (also known as current–voltage plot). Hence, the *I*–*V* scanning in potentiostatic or galvanostatic mode constitutes the basic characterization technique to explore the potential of new materials and cell structure design. Similarly, as the stress–strain curve of the standard samples was employed to reflect the basic mechanical properties of reported structural materials as shown in Figure 1a–c, the polarization curve can be widely employed to characterize the electrical properties of SOFCs (shown in Figure 1d–f). Naturally, searching for a standard test method to obtain comparable power results can be beneficial to promote the new developments in LTSOFCs.

The lack of an industry-level standard to acquire the polarization curve as its counterpart PEMFC, hampers the effective comparison of peak power density data of LTSOFC from various laboratories and institutions. A possible solution to tackle this predicament is to introduce a standard procedure of testing cells under an identical condition. This issue has been realized gradually and new testing procedures with a high standard have been proposed based on the single component fuel cells (SCFCs) [11]. The possible factors have been summarized from two aspects: geometric size and temperature. The constructed cells with a diameter of 13–30 mm are introduced in the experimental labs for fuel cell measurements. For the limited constructed cell size (13 mm for instance), just a 1 mm error (by sample loading/or fabrication) would lead to at least a 17% non-determinacy by directly affecting the current and power output of the testing cell. In addition, uncertainty in operational temperature fluctuation (even a 20 °C fluctuation of cell temperature) may lead to a fluctuation of at least a 10% variation on ionic conductivity of the cell, thereby reducing the practical performance. As a result, this a framework about cells fabrication and standard experiment procedures has been listed for SCFCs.

Though the aforementioned strategy of statistic duplicate verification via meticulous experiments can slightly increase the confidence level of the power output-measured polarization curves, there is still inadequate information between the transient state and steady state performance of LTSOFCs. The traditional solid oxide cells (those adopting single SDC or GDC thin electrolyte), which are usually subjected to high-temperature calcination, are more or less non-consistent in their current–voltage characteristic and short-term duration performance in terms of output power. Furthermore, the achieved peak power results are higher than the derived data from stability curves [12,13]. In particular, remarkable derivation is a common phenomenon of SIFC or SMFC, which may be attributed to its inherent electronic conduction and non-dense EFL layer structures. 

In the first section of the case study, we will demonstrate that such derivation cannot be eliminated by repeating the experiments for current–voltage curves, which may result from the transient state of test cells instead of the steady state. Steady state means the voltage of cells corresponding to the specific current did not vary with the time [14]. With regard to transient state, the voltage of the cell needed several seconds/minutes/hours to reach a stable point affected by the time constant, which is further correlated with the cell materials/structure and thermal history. If the transient state remains constant during the current–voltage sweeping, the recorded power output of LTSOFC will increase with the increased scanning rate/speed.

The valuable indicators of fuel cell characteristics (power density and enhanced ionic conductivity) rely on the polarization/*I*–*V* curve. Therefore, we attempted to establish a modified standard procedure for assessing a reliable polarization curve with improved confident level (CL), namely a diagram conversion from transient to steady state evaluation. 

## 2. Case Study

Currently, research seldom provides complete testing conditions and whole parameters of polarization curves as reported by Rauf et al. [15], such as hydrogen flux, cells thermal history, device fabrication, and scanning method. Among them, the current–voltage sweeping rate/speed is previously considered as an insignificant factor, whereas it indeed has a great influence on the final curve profile. Based on the previous literature, most PPD/MPD data are based on transient polarization curves, and the aforementioned deviation between the transient and steady state performance in LTSOFCs is ubiquitous [14]. The NSDC-LCCN nanocomposite electrolyte is a recently reported work that illustrates this phenomenon [16]. As shown in Figure S1a, 3NSDC-LCCN cells exhibit a high apparent peak power density of about 600 mW/cm^2^ at 550 °C. The obtained 0.81 V operating voltage corresponds to the current density of 469 mA/cm^2^ under fuel cell conditions. While, the operating voltage of cell can only reach 0.45 V at the identical current density according to the steady-state curve (Figure S1b). Obviously, cells present transient state characterization during fast current–voltage sweeping/scanning way. In addition, this transient state will lead to significant discreteness in power output.

In the current–voltage/polarization curve measurements of constructed NSDC-LCCN cells, as well as previous reported results, demonstrated high power density in SIFC and SMFC [17,18]. The parameters, with a step size/voltage interval of 0.01–0.02 V and a time interval of 0.5 s were utilized during *I*–*V* sweeping with a short circle of 1–2 min. Thus, the power density data might be a transient state result for the cells subjected to fast sweeping/scanning rate. In comparison, the representative sweeping rate of 1 mV/s is usually employed in the YSZ, conventional doped ceria and LSGM electrolyte cell, in the electro-chemical workstation, which further corresponds to *I*–*V* measurements for 10–15 min. It is reasonable to believe that the cell exhibits an apparent power density (APP) instead of stable power density (SPP) in the instantaneous/quick polarization curve measurements, and transient state characteristics are beyond the actual operating performance of fuel cells. 

**Figure 2:**
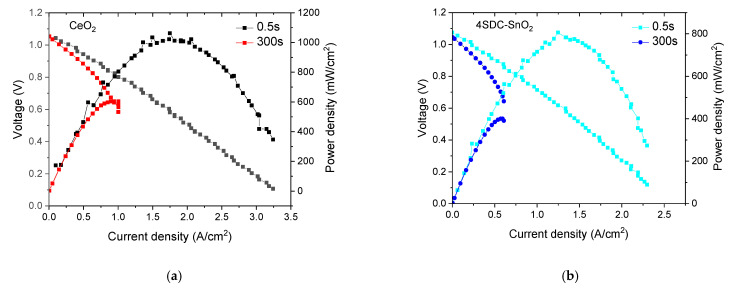
*I*–*V*–*P* curve of cells by sweeping on different time intervals (**a**) CeO_2_; (**b**) 4SDC-SnO_2_ electrolyte.

To further illustrate the great influence of sweeping/scanning rate on the apparent power density of SJFC/SMFC, *I*–*V*–*P* curves were demonstrated. For example, the reported high oxide ion-conducting electrolytes, such as non-doped ceria (CeO_2_) [19,20] and SDC-SnO_2_ [21] heterostructure have fast ionic transportation as well as high power density at low operational temperature. In addition, the fabricated devices with schematic PEN structure, i.e., CeO_2_ and SDC-SnO_2_ electrolytes sandwiched with identical symmetrical NCAL electrode, are fabricated (Figure S2). The measurement procedure of polarization curves and involved devices setup are ascribed in the supplemental data. Moreover, the comparative exploration of polarization curves in potentiostatic mode has been conducted by undergoing a quick scan of 0.5 s time interval. A slower scan with a 300 s time interval was set, in which the latter can be regarded as a quasi-steady-state characterization due to the notably reduced sweeping rate of about 0.06 mV/s during current–voltage measurement (around a half of 1 m/V). As shown in Figure 2, the constructed cell with commercial CeO_2_ as electrolyte membrane displayed a distinguished MPD/PPD over 1000 mW/cm^2^ at 550 °C (0.5 s case). Nevertheless, in the case of the 300 s time interval, the polarization curve shifted down and left with an obvious drop of 500 mW/cm^2^ because of the transient to steady-state characteristics. Similarly, the peak power density of 4SDC-SnO_2_ declined by half with the increase of time interval. It is also worthy to note that the quasi-steady-state polarization curves of CeO_2_ and 4SDC-SnO_2_ cells exhibited a special asymmetric and default feature, in which the *I*–*V* profile is cut-off after peak power point because of the reverse output current at constant potential (as shown in Figure S3). In galvanostatic measurement mode, the cell voltage exhibited a catastrophic instability by entering of concentration polarization zone.

## 3. Standard Test Procedure

Generally, SOFCs need a certain time to activate and reach a steady state. In this respect, extending the testing time consumption is a simple way to approach steady state evaluation. Therefore, trading off between economic and technical acceptability is the crucial issue for the standard procedure of acquiring a reliable polarization curve, in which suitable time interval should be sought in the current–voltage sweeping/scanning instead of fast measurements in the transient state approach.

**Figure 3:**
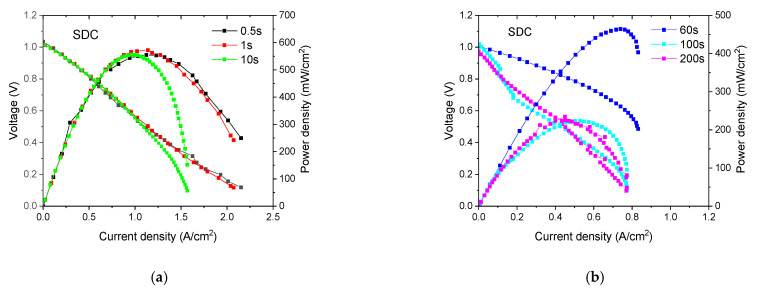
The peak performance curve of SOFCs using SDC electrolyte with (**a**) 0.5–10 s time interval; (**b**) 60–200 s time interval.

For primary screening, LTSOFCs using conventional SDC electrolyte were studied as a prototype cell, in which the SDC was prepared by co-precipitation method using sodium carbonate (Na_2_CO_3_) as a precipitating agent. The derived current–voltage sweeping curves are shown in Figure 3, with time intervals of 0.5 s. 1 s, 10 s, 60 s, 100 s and 200 s, respectively. It was easily figured out that the SDC cell power density decreases with the increase of time interval, which is quite similar to non-doped CeO_2_ and SDC-SnO_2_ heterostructure. However, the capacity of completing the whole *I*–*V* profile without a cut-off effect in the quasi-steady-state evaluation, indicates that the Na_2_CO_3_-precipitated SDC may have a superior operative potential at high-current density than ammonium hydrogen carbonate (NH_4_HCO_3_)-precipitated SDC materials (employed in the SDC-SnO_2_ heterostructure sample), as shown in Figure 3b. In addition, the PPD/MPD results coincide with the steady value of around 200 mW/cm^2^ with a time interval of 100–200 s. Thus, 100 s is chosen as the standard time interval temporarily.

**Figure 4:**
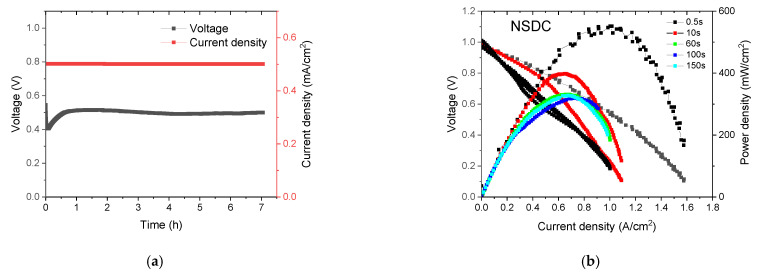
(**a**) Steady-state power output of NSDC cell; (**b**) Polarization curve with voltage interval 0.01 V and scanning time interval of 1 s, 10 s, 60 s, 100 s, and 200 s.

Another possible factor that has to be excluded is durability measurements, because the time duration of cells also contributes to analyzing the sweeping rate-dependence of the apparent power density. Hence, the further exploration on modified standard procedure of polarization curve should be based on a verified stable fuel cell system. To this end, NSDC nanocomposite electrolyte with short-term duration at considerable current-load as Ref. [16] was fabricated and measured at different sweeping rates (modulated by time interval). The NSDC cell was proven to be stable at operating current density of 0.5 A/cm^2^ as shown in Figure 4a. Moreover, it can be concluded from the series of polarization curves (Figure 4b) that the sweeping rate of *I*–*V* profile shows a limited effect on the final performance of the stable cell system. For the strongly transient state evaluation, as demonstrated by a quick sweeping in the case of 0.5 s, the cell exhibits the top apparent output power, peak at about 560 mW/cm^2^. Thus, the time interval extension effectively decreases the record power output to peak value of about 300 mW/cm^2^ with the difference between 100 s and 150 s of scanning.

According to the above investigation, a modified test procedure on constructed cell (lab-scale) by quasi-steady-state characteristic could be described as:

**Part I: Preparation process**

Stabilization of Cell includes the heat-up of test holder with button cell in furnace (setting at specific temperature), e.g., heating at 500–600 °C for 30 min, then inlet fuel (hydrogen)/air (oxygen) gas into anode and cathode, respectively, until the whole system reached a thermal equilibrium state with a fixed OCV.

**Part II: Measure process**

Undergo a standard current–voltage sweeping from OCV to 0.4 V below the threshold scanning rate/speed (1 mV/s).

For clarity, a schematic parameter set of the modified standard procedure of current–voltage characteristic is listed in Table 1. Among the three setups, P3 (0.01 V step size with time interval of 100/200 s) is believed to achieve the highest confidence level. Therefore, the polarization curve is a steady state characterizing technique, in which the cells have to fulfill the requirement of steady state, though the proposed standard procedural measurements already employed the lowest sweeping rate. To assess an actual steady state performance, one can adopt optimal stabilization process, in which the cell was operated in the activation zone, i.e., at around 100 mA/cm^2^ for 5–10 h before undoing *I*–*V* sweeping.

## 4. Conclusions

A standardized measurement process by acquiring reliable polarization curve of the constructed cell plays a pivotal role in SOFC/SIFC. Even small geometrical errors may cause huge differences in the electro-chemical properties. Consistent testing conditions are also meaningful for the screen out of promising systems.

Furthermore, this case study reveals that it is reasonable to infer that the conventional test procedure on transient state evaluation may induce an appreciable error in measuring electro-chemical properties. Exploring the power density data of cells with an improved confidence level can be facilitated by a modified quasi-steady state characterization of polarization curves, employing a current–voltage sweeping with sampling/time interval of 100 s. Compared with transient state evaluation, the normalized MPD from the updated standard procedure of *I*–*V* curve on quasi-steady state represents a better approximation of actual performance of fuel cells. The uncertainty of measurement of errors in various LTSOFC systems can also be expected to significantly lessened by the proposed approach.

## Figures and Tables

**Table 1:** A schematic parameter set of the proposed standard procedure of polarization cure measurement.

Content	P1	P2	P3
Device	Electrical chemical workstation	Electrical Load	Electrical Load
Sweeping parameter	0.001 V, 1 s	0.01 V, 60 s	0.01 V, 100/200 s
Scanning rate	1 mV/s	0.16 mV/s	0.05–0.1 mV/s
Single *I*–*V* scan	>15 min	>60 min	>80 min
Confidence Level (CL)	☆☆☆	☆☆☆☆	☆☆☆☆☆

## Data Availability

Data is contained within the article or supplementary material.

## Supplementary Materials

The following are available online at https://www.mdpi.com/article/10.3390/nano11081923/s1, Figure S1:(a) The polarization curve of NSDC-LCCN cell; (b) the duration of NSDC-LCCN cell (Permission and redrawn from Ref. [1]), Figure S2: Illustration of single fuel cell, Figure S3: Full I-V-P profile of cells by sweeping on different time intervals (a) CeO2; (b) 4SDC-SnO2 electrolyte.
