# Supplementary material for: Standardized Procedures Important for Improving Low-Temperature Ceramic Fuel Cell Technology: From Transient to Steady State Assessment
Source: Nanomaterials (Basel). 2021 Jul 26;11(8):1923. doi: 10.3390/nano11081923 (PMC8399102; doi:10.3390/nano11081923)
Supplement: Supplementary file 1 [file nanomaterials-11-01923-s001.zip › nanomaterials-1270755-supplementary.pdf]

Supplementary Materials

Standardized Procedures Important for Improving Low-Temperature Ceramic Fuel Cell Technology: From Transient to Steady State Assessment

Fan Yang [1,2,*,†], Yifei Zhang [1,†], Jingjing Liu [1], Muhammad Yousaf [1], Xinlei Yang [1]

[1] Jiangsu Provincial Key Laboratory of Solar Energy Science and Technology, School of Energy & Environment, Southeast University, Nanjing 210096, China; YFZH147@163.com (Y.Z.); liujingjing_970812@163.com (J.L.); tahayousaf@gmail.com (M.Y.); brobow@163.com (X.Y.)
[2] Key Laboratory of Energy Thermal Conversion and Control of Ministry of Education, School of Energy & Environment, Southeast University, Nanjing 210096, China
* Correspondence: yang_fan@seu.edu.cn or yangfan_hit@126.com
† These authors contribute equally to this work.

Synthesis of SDC and NSDC

Co-precipitation method was used to synthesis $Ce_{0.8}Sm_{0.2}O_{2-\delta}$ (SDC) sample. Stoichiometric amounts of $Ce(NO_3)_3{\cdot}6H_2O$ (Aladdin Bio-Chem Technology Ltd., Shanghai, China) and $Sm(NO_3)_3{\cdot}6H_2O$ (Aladdin Bio-Chem Technology Ltd., Shanghai, China) were dissolved in deionized water with a concentration of 0.5M. Meanwhile, Na_2CO_3 (Aladdin Bio-Chem Technology Ltd., Shanghai, China) and NH_4HCO_3 (Aladdin Bio-Chem Technology Ltd., Shanghai, China, 1M) were selected as the precipitating agent, respectively. According to the metal ion: carbonate ion (1:1.5 molar ratio), Na_2CO_3 or NH_4HCO_3 solution was dropwise added into the Nitrate solution with the continuous stirring for 4h. Next step, the solution was filtered out after 3 times washing with deionized water. Then, the acquired precipitates were dried for at 120°C 12h in heating/drying oven. Finally, the dried precipitates were calcined at 800°C for 4h into the muffle furnace.

NSDC powder was synthesized by simple solid state method. As-prepared SDC powder (Na_2CO_3 as the precipitation agent) was mixed with Na_2CO_3 with the weight ratio of SDC:Na_2CO_3 = 4:1. After that, ethanol was dropwise added into the mixed powders and grinded for 1h. Finally, the mixed materials were calcined at 750°C for 4h to obtain NSDC.

Synthesis of SDC-SnO2

Simple solid state method was also used for the fabrication of SDC-SnO_2. SDC (NH_4HCO_3 as the precipitation agent) and SnO_2 (Aladdin Bio-Chem Technology Ltd., Shanghai, China) were mixed with the mass ratio of 4:1. Then the mixture was grinded with ethanol solution for 1h to form SDC-SnO_2 precursor. Finally, the precursors were calcined for 3h at 750°C to obtain SDC-SnO_2 composites.

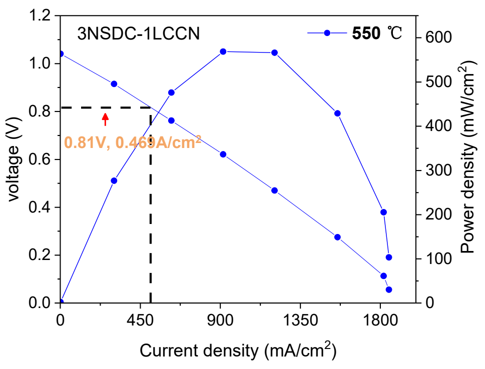

(a)

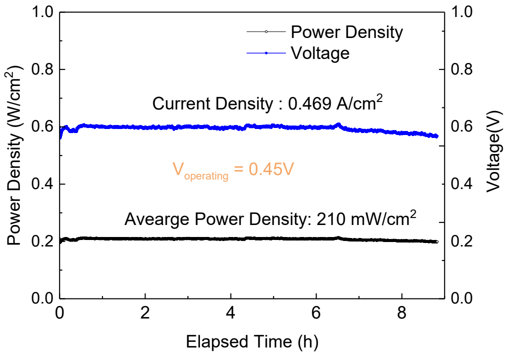

(b)

Figure S1. (a) The polarization curve of NSDC-LCCN cell; (b) the duration of NSDC-LCCN cell (Permission and redrawn from Ref. [1])

Standard test procedure section

First, for the measurement of constructed fuel cell device like fig. S2, it is recommended to set the diameter of device as 13mm and unify the effective area of 0.64cm^2. 220Mpa pressure is normally used to press the cell (keep 1min), which usually contains 0.3g powder for electrolyte. Further, the fabricated cell is put into heating oven and preheated for 45min at constant temperature. Regardless of the geometry, hydrogen flux and air flow are both crucial factors, which will have a great influence on the performance of fuel cells. So, hydrogen flux is set at 150ml/min and air flow was 300ml/min.

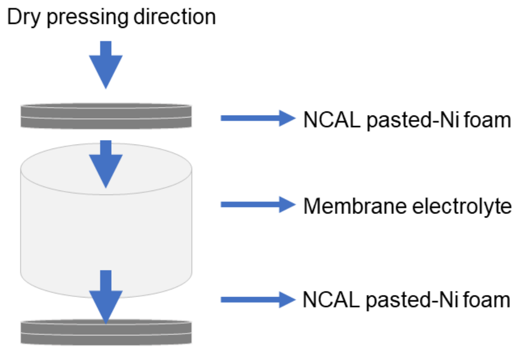

Figure S2. Illustration of single fuel cell

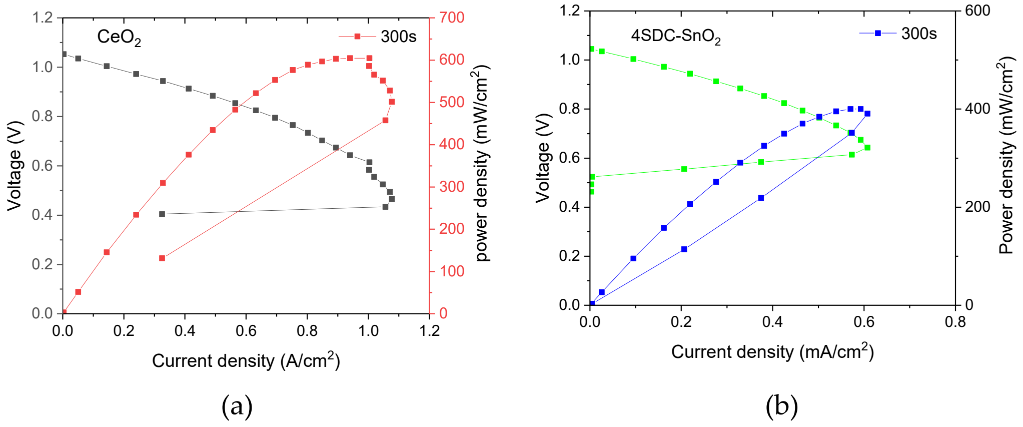

Figure S3. Full I-V-P profile of cells by sweeping on different time intervals (a) CeO_2; (b) 4SDC-SnO_2 electrolyte.

References:

1. Yang F, Dong T, Zhang X, Liu J, Tian W, Zhang Y. Semiconductor ionic Ce0.8Sm0.2O2-δ-Na2CO3 – LiCo0.225Cu0.075Ni0.7O3-δ composite material as electrolyte for low temperature solid oxide fuel cells. INT J HYDROGEN ENERG. 2020, 45, 14972-8.
